# Adherence to rivaroxaban versus apixaban among patients with non-valvular atrial fibrillation: Analysis of overall population and subgroups of prior oral anticoagulant users

**DOI:** 10.1371/journal.pone.0194099

**Published:** 2018-04-05

**Authors:** Colleen A. McHorney, Concetta Crivera, François Laliberté, Guillaume Germain, Willy Wynant, Patrick Lefebvre

**Affiliations:** 1 Evidera, Bethesda, Maryland, United States of America; 2 Janssen Scientific Affairs, LLC, Raritan, New Jersey, United States of America; 3 Groupe d’analyse, Ltée, Montréal, Québec, Canada; Maastricht University Medical Center, NETHERLANDS

## Abstract

**Background:**

Medication non-adherence can result in poor health outcomes. Understanding differences in adherence rates to non-vitamin K oral anticoagulants (NOACs) could guide treatment decisions and improve clinical outcomes among patients with non-valvular atrial fibrillation (NVAF).

**Objective:**

To compare adherence to rivaroxaban and apixaban among the overall NVAF population and subgroups of prior oral anticoagulant (OAC) users (e.g., multiple comorbidities, non-adherence risk factors).

**Methods:**

Using healthcare claims from the Truven Health Analytics MarketScan (7/2012-7/2015), adult patients with ≥2 dispensings of rivaroxaban or apixaban ≥ 180 days apart with > 60 days of supply, ≥ 6 months of pre- and post-index eligibility, ≥ 1 atrial fibrillation diagnosis pre- or on the index date, and without valvular involvement were identified. Propensity-score methods adjusting for potential baseline confounders were used to create matched cohorts of rivaroxaban and apixaban patients. Adherence was assessed during the implementation phase using the percentage of patients with proportion of days covered (PDC) ≥0.8 at 6 months. Subgroups of patients with prior OAC use were evaluated; additional subgroups were identified and evaluated by Quan-Charlson Comorbidity index ≥2 and presence of non-adherence risk factors (i.e., mental disorders, stress, isolation, and rheumatoid arthritis).

**Results:**

A total of 13,890 NVAF subjects were included in each of the 2 matched cohorts. All baseline characteristics were balanced between cohorts. At 6 months, significantly more rivaroxaban users were adherent to treatment compared to apixaban users (81.8% vs. 78.0%; absolute difference of 3.8%; p<.001). Rivaroxaban users had significantly higher adherence rates in all subgroups examined.

**Conclusion:**

Rivaroxaban users had consistently higher adherence rates than apixaban users overall and among all NVAF subgroups examined.

## Introduction

Atrial fibrillation (AF) patients are at increased risk of stroke [1–3] and often require long-term preventive treatment with anticoagulants. [4,5] Anticoagulation is highly effective for stroke prevention in AF. [6,7] However, to benefit from the treatment, optimal patient adherence to medication is crucial. This is especially true with non-vitamin-k antagonist oral anticoagulants (NOACs)–a new generation of oral anticoagulants (OACs) that, unlike the previous standard of care of warfarin, have a short half-life (12 hours or less). [8] NOACs offer a life-style improvement compared to warfarin due to fewer interactions with food and other medications, a stable dosing regimen, and lack of routine laboratory monitoring. NOAC’s less burdensome treatment can translate into better adherence. However, the lack of regular monitoring has raised concerns that some patients may have difficulties with remembering to take their medications [9] or exhibit other intentional or unintentional adherence barriers.

Understanding adherence to different NOACs can yield information about their comparative advantages and assist physicians and patients with choosing the best treatment strategy. It is well established that simplified dosing regimens improve adherence. [10–13] In the context of NOACs, a recent study reported that, based on the Pharmacy Quality Alliance (PQA) adherence measure, [14] patients receiving rivaroxaban administered once daily (QD) had better adherence compared to patients receiving apixaban administered twice daily (BID).[15]

Since NVAF OACs are preventive treatments that do not address any symptoms, patient motivation to take the medication properly, especially among new users, could be fragile. Therefore, this study focused on a comparison of adherence between patients treated with rivaroxaban or apixaban in a population of NVAF patients using observational insurance claims data. It was hypothesized that prior OAC users would have more experience with NVAF, better knowledge about the therapy and its benefits, and, thus, higher motivation to take the medication as prescribed. Among these more experienced and potentially more motivated patients, additional stratified analyses were conducted to compare how presence of multiple comorbidities (including some specific non-adherence risk factor for NVAF patients: age, history of diabetes and hypertension) [16] as well as cognitive and functional risk factors for non-adherence are associated with rivaroxaban and apixaban intake.

## Methods

### Data source

Claims from the Truven Health Analytics MarketScan databases (7/2012-7/2015) were used for this study. These longitudinal databases include the Commercial Claims and Encounters database and the Medicare Supplemental and Coordination of Benefits database which combine claims from approximately 100 payers (including commercial insurance companies, Blue Cross and Blue Shield plans, and third-party administrators). The data come from a selection of large employers, health plans, and government and public organizations and represent roughly 75 million covered lives in the most recent full data year (including employees and their dependents, self-insured employers, and Medicare-eligible retirees with employer-provided Medicare supplemental plans). The databases cover all census regions of the US with concentration in the South and North Central (Midwest) regions. The databases are fully compliant with the Health Insurance Portability and Accountability Act of privacy.

### Study design

The study had a retrospective matched cohort design. Adult patients were included if they had at least 2 dispensings of the same NOAC (i.e., rivaroxaban or apixaban) at least 180 days apart after the approval date of apixaban (December 28, 2012). Patients with more than 60 days of supply of the same agent (rivaroxaban or apixaban) were selected (consistent with the PQA-endorsed adherence measure in chronic users of NOAC [14]) and patients with their index date before February 1, 2013 were excluded. These criteria were used to select chronic NOAC users (i.e., patients that are using long-term anticoagulation therapy). Of note, patients who received procedures aimed to cure NVAF (i.e., catheter ablation and maze surgery; ICD-9-CM: 37.34, CPT-4: 93650–93652, 93799, 33250–33251, 33254–33259, 33261, 33265–33266) were censored at the date of the procedure which could affect the above inclusion criteria.

The start date of the first dispensing was termed as the index date and the drug dispensed on that day was termed as the index drug. Patients were required to have at least 6 months of continuous health plan enrollment pre- and post-index, and at least one diagnosis of AF (International Classification of Diseases, Ninth Edition-Clinical Modifications [ICD-9-CM] code 427.31) during the 6-month period preceding the index date (baseline period) or on the index date. NVAF diagnoses were identified using medical claims and required the absence of a mitral-stenosis (ICD-9-CM: 394.0x, 394.2x, 396.0x, 396.1x, 746.5x, 996.02, 996.71), mechanical heart-valve (ICD-9-Proc: 35.20, 35.22, 35.23, 35.24, 35.97; CPT-4: 33405, 33430, 33420, 33422, 33425–33427, 92987), or organ/tissue replacement by transplant (i.e., heart valve: ICD-9-CM: V42.2) in patient history during the baseline period. In addition, patients who had dispensings for the index drug or lived in assisted care facilities during the 6-months baseline period or were pregnant during the baseline period or the observation period (i.e., 6 months post index date) were excluded.

Subgroups of patients were created as follows: 1) prior OAC users (i.e., patients who used warfarin or a non-index NOAC during the baseline period) vs. non-prior OAC users; and prior OAC users were further broken down by 2) presence or absence of multiple comorbidities defined as baseline value of Quan-Charlson comorbidity index (QCCI) ≥ 2); and 3) presence or absence of non-adherence risk factors defined as mental disorders (i.e., depression, schizophrenic disorder, bipolar, anxiety, obsessive disorder, dissociative and depersonalization disorder, somatization disorders, feeding disorders, development disorders, substance abuse, sleeping disorders, neurocognitive disorders, medications, and personality disorders; S1 Table), stress, isolation, or rheumatoid arthritis (ICD-9 codes for non-adherence risk factors are presented in S1 Table). The choice of non-adherence risk factors was based on conditions that could interfere with patient cognitive or functional ability to take oral prescription medications. [17]

Patient characteristics were summarized during the 6-month baseline period preceding the index date and adherence was evaluated at 6 months post index date.

### Statistical analysis

#### Matching on baseline characteristics

Patient baseline characteristics were described using mean and standard deviation (SD) for continuous variables and frequency counts and proportions for categorical variables. To ensure that patients in the rivaroxaban and the apixaban cohorts had balanced characteristics at baseline, propensity-score matching was performed. Apixaban patients were matched 1:1 to rivaroxaban patients using propensity score categories of 25%. Variables used in the propensity-score estimation were the following: age, sex, region, insurance type, month and year of index date, individual risk factors for stroke and bleeding, presence of venous thromboembolism (VTE), total hip and total knee arthroplasty (THA/TKA), mail-order pharmacy for index medication, use of dabigatran or warfarin at baseline, QCCI, CHA_2_DS_2_-VASc score, and HAS-BLED score. Baseline characteristics were compared for unmatched and matched cohorts using standardized differences with a threshold of 10% to assess sufficient balance between covariates. [18]

#### Adherence measure

Adherence to the index anticoagulant (apixaban or rivaroxaban) was measured among chronic NOAC users with the proportion of days covered (PDC) over the first 6 months following the index date. The PDC was calculated for each patient as the total number of days of supply for the index anticoagulant agent divided by a specific period of time which was 6 months in this study. The 6-month period post-index was used to ensure that all matched patients were available when the adherence measure was evaluated to avoid right censoring. Adherence to treatment was defined using a PDC ≥ 0.8 threshold as recommended by the PQA. [19] The PQA measurement and development process is underlain by a consensus-driven approach involving experts in all phases of drug use and management. This led to the development and endorsement of several medication quality measures. A sensitivity analysis was also conducted using a more conservative threshold of PDC ≥ 0.9 to define adherence.

In this analysis, a dispensing was considered an “early refill” if it was dispensed within 7 days of the run-out date of the current dispensing (i.e., dispensing date plus the days of supply). In this case, the second dispensing was moved to the end of the first dispensing and was treated as an early refill by the patient (we assumed that the patients took the previous dispensing entirely before starting the new one). Days of supply of a non-index anticoagulant agent in patients who switched during the first 6 months of the observation period were not counted in the PDC of the index drug given that the focus in this study concerned adherence to particular therapies.

With respect to the taxonomy of adherence (i.e., initiation, implementation, discontinuation) established by Vrijens et al. [20], the PQA methodology was used in the present study to evaluate the implementation phase of adherence. More specifically, all patients included in the current study had at least two dispensings of the same NOAC agent 180 apart, and the PDC was evaluated over the first 6 months of observation. Therefore, all patients should have been using their medication during these first 6 months of observation.

## Results

**Fig 1:**
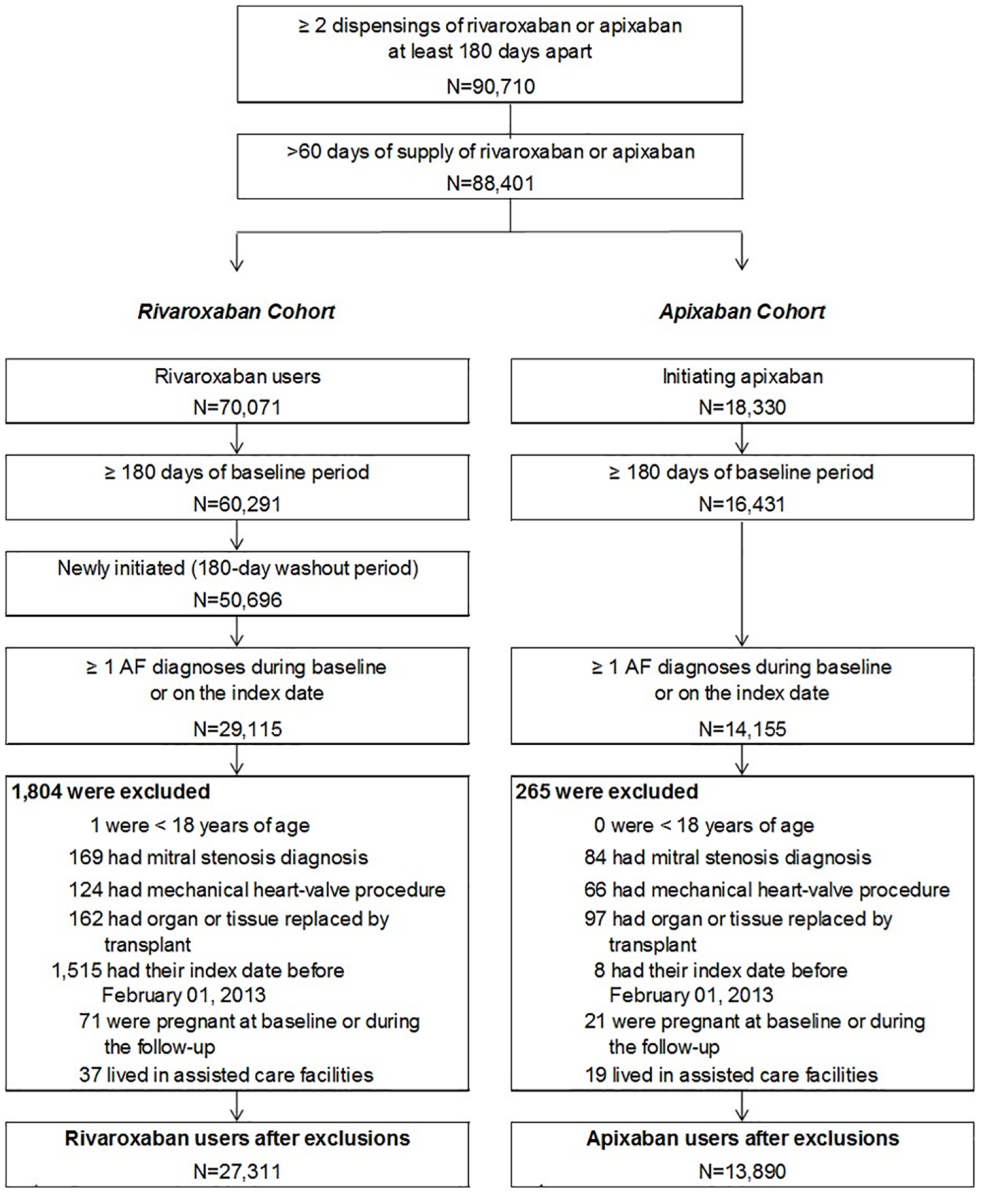
Sample selection. AF: atrial fibrillation.

A total of 90,710 patients were identified with ≥2 dispensings of rivaroxaban or apixaban at least 180 days apart in the database. After applying the inclusion and exclusion criteria, 27,311 and 13,890 patients were identified in the rivaroxaban and apixaban cohorts, respectively (Fig 1). Before matching, rivaroxaban users were, on average, only one year younger (mean age 70.0 versus 71.3) compared to apixaban users. Apixaban patients had slightly higher QCCI (1.81 versus 1.69), CHA_2_DS_2_-VASc score (3.52 versus 3.36), and HAS-BLED score (1.81 versus 1.70) compared to rivaroxaban before matching. In addition, the prevalence of VTE at baseline was higher (7.5% versus 2.9%) in rivaroxaban compared to apixaban patients. About 31% of patients in both cohorts used a non-index OAC (most commonly warfarin) during the baseline period. The proportion of patients with non-adherence risk factors was similar in the rivaroxaban cohort (32.7%) compared to the apixaban cohort (33.8%) as were the number of patients with >2 risk factors for non-adherence (6.6% and 6.0% for rivaroxaban and apixaban patients, respectively; Table 1). Following matching, all baseline characteristics were well-balanced between cohorts (standardized differences all <10%), and each treatment cohort included 13,890 patients.

**Table 1:** Patient baseline characteristics.

Characteristics	Un-Matched Cohorts			Matched Cohorts^1^		
	Rivaroxaban	Apixaban	Std Diff (%)^2^^,^^3^	Rivaroxaban	Apixaban	Std Diff (%)^2^^,^^3^	
	(N = 27,311)	(N = 13,890)		(N = 13,890)	(N = 13,890)		
**Demographics**^4^						
Age, mean [median] (SD)	70.03 [71] (11.6)	71.27 [72] (11.6)	10.7	71.04 [72] (11.4)	71.27 [72] (11.6)	2.0
Gender, female, n (%)	11,201 (41.0)	5,910 (42.5)	3.1	5,914 (42.6)	5,910 (42.5)	0.1
**Region**^5^, **n (%)**						
South	9,225 (33.8)	5,060 (36.4)	5.6	5,017 (36.1)	5,060 (36.4)	0.6
West	3,464 (12.7)	1,546 (11.1)	4.8	1,552 (11.2)	1,546 (11.1)	0.1
North Central	7,963 (29.2)	4,024 (29.0)	0.4	4,059 (29.2)	4,024 (29.0)	0.6
Northeast	6,301 (23.1)	3,089 (22.2)	2.0	3,103 (22.3)	3,089 (22.2)	0.2
Unknown	358 (1.3)	171 (1.2)	0.7	159 (1.1)	171 (1.2)	0.8
**Insurance type**^4^, **n (%)**						
PPO	13,434 (49.2)	6,665 (48.0)	2.4	6,819 (49.1)	6,665 (48.0)	2.2
HMO	2,077 (7.6)	959 (6.9)	2.7	938 (6.8)	959 (6.9)	0.6
Comprehensive	8,648 (31.7)	4,697 (33.8)	4.6	4,559 (32.8)	4,697 (33.8)	2.1
POS	1,406 (5.1)	627 (4.5)	3.0	647 (4.7)	627 (4.5)	0.7
CDHP	851 (3.1)	502 (3.6)	2.8	483 (3.5)	502 (3.6)	0.7
EPO	107 (0.4)	46 (0.3)	1.0	45 (0.3)	46 (0.3)	0.1
POS capitated	124 (0.5)	34 (0.2)	3.5	37 (0.3)	34 (0.2)	0.4
HDHP	373 (1.4)	214 (1.5)	1.5	216 (1.6)	214 (1.5)	0.1
Not specified	291 (1.1)	146 (1.1)	0.1	146 (1.1)	146 (1.1)	0.0
**Comorbidity index scores**^5^						
QCCI, mean [median] (SD)	1.69 [1] (1.9)	1.81 [1] (2.0)	6.1	1.75 [1] (2.0)	1.81 [1] (2.0)	3.0
QCCI ≥2, n (%)	11,361 (41.6)	6,178 (44.5)	5.8	5,979 (43.0)	6,178 (44.5)	2.9
CHA_2_DS_2_-VASc, mean [median] (SD)	3.36 [3] (1.9)	3.52 [3] (1.9)	8.4	3.47 [3] (1.9)	3.52 [3] (1.9)	2.4
HAS-BLED, mean [median] (SD)	1.70 [2] (1.0)	1.81 [2] (1.0)	11.4	1.77 [2] (1.0)	1.81 [2] (1.0)	3.5
**Comorbidities**^5^, **n (%)**						
VTE	2,057 (7.5)	409 (2.9)	20.6	519 (3.7)	409 (2.9)	4.4
THA/TKA	508 (1.9)	136 (1.0)	7.4	142 (1.0)	136 (1.0)	0.4
**Bleeding and stroke risk factors**^5^^,^^6^, **n (%)**						
Hypertension	18,649 (68.3)	10,021 (72.1)	8.4	9,882 (71.1)	10,021 (72.1)	2.2
Diabetes	7,634 (28.0)	4,008 (28.9)	2.0	3,918 (28.2)	4,008 (28.9)	1.4
Cerebrovascular accident (stroke)	4,019 (14.7)	2,245 (16.2)	4.0	2,128 (15.3)	2,245 (16.2)	2.3
**Bleeding risk factors**^5^^,^^6^, **n (%)**						
Anemia	3,297 (12.1)	1,819 (13.1)	3.1	1,747 (12.6)	1,819 (13.1)	1.5
Renal disease	3,255 (11.9)	2,066 (14.9)	8.7	1,913 (13.8)	2,066 (14.9)	3.1
Excessive fall risk	2,798 (10.2)	1,405 (10.1)	0.4	1,391 (10.0)	1,405 (10.1)	0.3
Chronic kidney disease	2,448 (9.0)	1,651 (11.9)	9.6	1,499 (10.8)	1,651 (11.9)	3.5
**Stroke risk factors**^5^^,^^6^, **n (%)**						
Hyperlipidemia	13,644 (50.0)	7,433 (53.5)	7.1	7,317 (52.7)	7,433 (53.5)	1.7
Coronary heart disease	9,331 (34.2)	5,338 (38.4)	8.9	5,213 (37.5)	5,338 (38.4)	1.9
Heart failure	5,280 (19.3)	2,980 (21.5)	5.3	2,890 (20.8)	2,980 (21.5)	1.6
Anti-hypertensive medication	4,244 (15.5)	2,856 (20.6)	13.1	2,625 (18.9)	2,856 (20.6)	4.2
COPD	3,393 (12.4)	1,742 (12.5)	0.4	1,717 (12.4)	1,742 (12.5)	0.5
Obesity	2,975 (10.9)	1,517 (10.9)	0.1	1,482 (10.7)	1,517 (10.9)	0.8
**Baseline use of OACs**^5^						
Use of oral anticoagulant						
Any oral anticoagulant	8,602 (31.5)	4,292 (30.9)	1.3	4,329 (31.2)	4,292 (30.9)	0.6
Warfarin	6,454 (23.6)	3,105 (22.4)	3.0	3,190 (23.0)	3,105 (22.4)	1.5
Dabigatran	2,275 (8.3)	1,221 (8.8)	1.6	1,203 (8.7)	1,221 (8.8)	0.5
Rivaroxaban	0 (0.0)	17 (0.1)	4.9	0 (0.0)	17 (0.1)	4.9
Number of different drug classes	7.32 [7] (4.0)	7.71 [7] (3.9)	9.8	7.43 [7] (4.0)	7.71 [7] (3.9)	7.1
Mail-ordered pharmacy (index medication)	3,811 (14.0)	2,528 (18.2)	11.6	2,401 (17.3)	2,528 (18.2)	2.4
**Non-adherence risk factors, n (%)**^5^^,^^7^						
0	18,380 (67.3)	9,201 (66.2)	2.2	9,287 (66.9)	9,201 (66.2)	1.3
1	7,129 (26.1)	3,850 (27.7)	3.6	3,715 (26.7)	3,850 (27.7)	2.2
≥2	1,802 (6.6)	839 (6.0)	2.3	888 (6.4)	839 (6.0)	1.5

PPO: Preferred provider organization; HMO: Health maintenance organization; POS: Point-of-service; CDHP: Consumer directed health plan; EPO: Exclusive provider organization; HDHP: High-deductible health plan; QCCI: Quan-Charlson comorbidity index; AF: atrial fibrillation; THA/TKA: total hip arthroplasty and total knee arthroplasty; Std Diff: standardized difference; COPD: chronic obstructive pulmonary disease; OAC: oral anticoagulant.

Notes:

^1.^ Apixaban patients were matched 1:1 with rivaroxaban patients using propensity score calipers of 25%. Variables used in the propensity score calculation included the following: age, gender, region, insurance type, index month, baseline risk factors for stroke and bleeding, presence of venous thromboembolisms, total hip arthroplasty, total knee arthroplasty, use of other oral anticoagulants at baseline, QCCI, CHA2DS2-VASc score, and HAS-BLED score, and atrial fibrillation at baseline, and mail-ordered pharmacy of thet index medication.

^2.^ For continuous variables, the standardized difference is calculated by dividing the absolute difference in means of the apixaban and the rivaroxaban cohorts by the pooled standard deviation of both groups. The pooled standard deviation is the square root of the average of the squared standard deviations.

^3.^ For categorical variables with 2 levels, the standardized difference is calculated using the equation below where P is the respective proportion of participants in each group: (Papixaban-Privaroxaban)/√[p(1-p)], where p = (Papixaban+Privaroxaban)/2.

^4.^ Evaluated at the index date.

^5.^ Evaluated during the 6-month baseline period.

^6.^ Additional matching stroke and bleeding risk factors (i.e., frequency below 10%) not reported in this table include nondependent abuse of drugs, hepatic disease, left ventricular dysfunction, hip, pelvis, or leg fracture, thrombocytopenia (low platelet count), ETOH abuse, peptic ulcer, previous falls, fracture of radius and ulna, bleeding diathesis, depression, smoking, left ventricular hypertrophy, claudication, and family history of CVD. All these additional matching stroke and bleeding risk factors were well balanced, with standardized differences below 10%.

^7.^ Non-adherence risk factors include the following: mental disorders, substance abuse, isolation, stress, and rheumatoid arthritis

**Fig 2:**
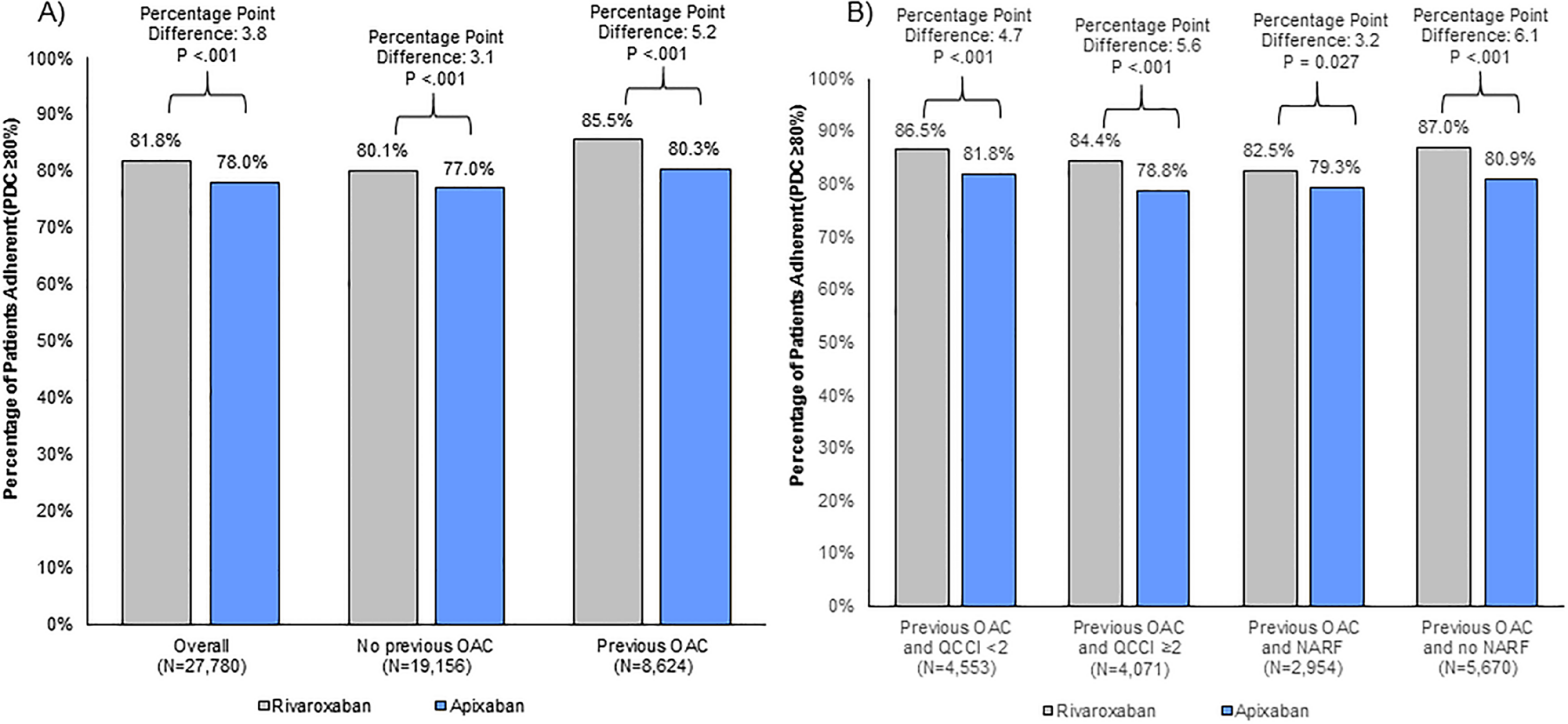
Adherence (PDC ≥0.8) difference in rivaroxaban compared to apixaban users at 6 months. (A) among all patients. (B) among patients with prior OAC use. OAC: oral anticoagulant; QCCI: Quan-Charlson Comorbidity Index; NARF: Non-adherence risk factors.

**Fig 3:**
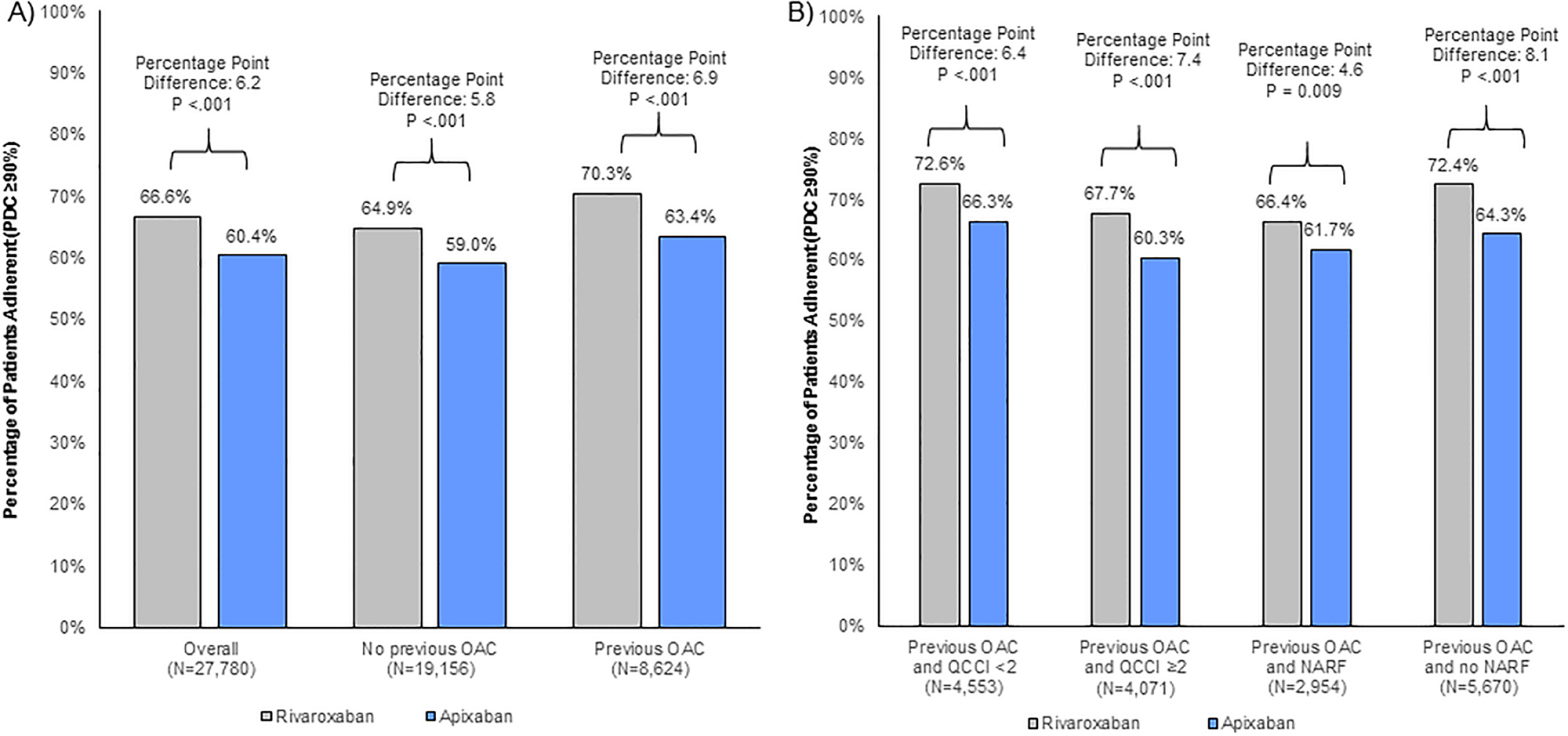
Adherence (PDC ≥0.9) difference in rivaroxaban compared to apixaban users at 6 months. (A) among all patients. (B) among patients with prior OAC use. OAC: oral anticoagulant; QCCI: Quan-Charlson Comorbidity Index; NARF: Non-adherence risk factors.

Results of the implementation phase of adherence were reported in Figs 2 and 3. The mean PDC was 88% and 86% for the rivaroxaban and apixaban cohorts, respectively (p<.001). Overall, significantly more rivaroxaban users were adherent to treatment compared to apixaban users (81.8% versus 78.0%; absolute difference of 3.8 percentage points; p<.001; Fig 2A). Among patients with no prior OAC use, the mean PDC was 87% and 86% for the rivaroxaban and apixaban cohorts, respectively (p<.001) and the proportion of adherent patients treated with rivaroxaban (80.1%) was higher compared to apixaban (77.0%; absolute difference: 3.1 percentage points; p<.001).

Adherence in the subgroup of prior OAC users was higher for both drugs in terms of mean PDC (90% and 88% for rivaroxaban and apixaban, respectively, p<.001), and the proportion of patient adherent ranged between 85.5% in rivaroxaban and 80.3% in apixaban patients with an absolute difference of 5.2 percentage points (p<.001) in favor of rivaroxaban (Fig 2A). Among prior OAC users with multiple comorbidities (QCCI ≥2), the mean PDC was 89% and 87% (p<.001) in the rivaroxaban and apixaban cohorts, while 84.4% and 78.8% of rivaroxaban and apixaban patients, respectively, were adherent (absolute difference of 5.6 percentage points; p<.001; Fig 2B). Among prior OAC users without multiple comorbidities (QCCI <2), a statistically significant difference was found in mean PDC (90% and 88%; p<0.01) and proportion of adherent patients (86.5% and 81.8%, absolute difference of 4.7 percentage points; p<.001) between rivaroxaban and apixaban patients. Adherence among prior OAC users with non-adherence risk factors was 82.5% in rivaroxaban and 79.3% in apixaban patients comprising an absolute difference of 3.2 percentage points (p = .027) between the cohorts (mean PDC was 89% and 87%, respectively; p = .006). The highest adherence and the largest difference between the cohorts were found in the subgroup of prior OAC users without non-adherence risk factors: mean PDC was 90% and 88% (p<.001), respectively; while 87.0% of rivaroxaban and 80.9% of apixaban patients were adherent to the index treatment for an absolute difference of 6.1 percentage points (p<.001).

The difference in adherence between rivaroxaban and apixaban users for the overall population as well as subgroups of patients with and without prior OACs using a higher threshold (PDC≥ 0.9) to define patients’ adherence is presented below (Fig 3A). In the overall population, there were also significantly more rivaroxaban users who were adherent to treatment compared to apixaban users with an absolute difference of 6.2 percentage points (66.6% versus 60.4%; p<.001). Difference for patients with and without prior OAC use were in a similar range (absolute differences of 6.9 and 5.8 percentage points, respectively; Fig 3A). As with the preceding definition for adherence among the subset of patients with prior OAC use, differences in adherence between rivaroxaban and apixaban among subgroups of patients with multiple comorbidities and without NARF (Fig 3B) increased compared to the overall population (Fig 3A) with absolute differences ranging between 7.4 and 8.1 percentage points (both p<.001). For the subgroups of patients with prior OAC use and non-adherence risk factors or without multiple comorbidities, rivaroxaban users were more adherent to treatment compared to apixaban users (absolute differences of 4.6 and 6.4 percentage points, respectively; both p = .009; Fig 3B).

## Discussion

This retrospective, observational study using insurance claims compared adherence to rivaroxaban and apixaban overall and in different subgroups of prior OAC users during the implementation phase of adherence to these agents. At 6 months, significantly more rivaroxaban users were adherent to treatment compared to apixaban users in the overall population, and this advantage was preserved in all subgroups of prior OAC users. In addition, absolute differences between rivaroxaban and apixaban users were found to be higher with a more conservative definition of adherence (PDC>0.9).

Results yielded in the overall analysis of this study are consistent with prior research reporting an adherence advantage of rivaroxaban over apixaban based on administrative claims data and the PQA methodology. [15,21] Although one administrative-claims study suggested that adherence rates were higher with apixaban compared to other NOACs (rivaroxaban and dabigatran) and warfarin, [22] it did not utilize the PQA methodology, as it was the case in this study, and the analysis compared anticoagulant agents based on an unbalanced follow-up period which could well explain the observed differences in adherence results. A study of patients in a well-structured AF clinic with a PQA-similar methodology found no difference in adherence rates between rivaroxaban and apixaban. [23] The rates reported in this study were well above 90% for both rivaroxaban and apixaban cohorts suggesting that patient education, motivation, support, and follow-up systems put in place in the AF clinic led to almost perfect adherence regardless of the agent used.

The contribution of the current research was the comparative adherence analysis in prior OAC users who initiated either rivaroxaban or apixaban and used it chronically. The findings supported the initial hypothesis that adherence was higher in prior OAC users which could be potentially explained by more experience with NVAF management, better understanding of anticoagulation therapy and its benefits, and, therefore, higher motivation to take the treatment as prescribed. [24] In terms of the comparative advantages of rivaroxaban and apixaban among prior OAC users, rivaroxaban was associated with significantly better adherence with an absolute difference of 5.2 percentage points. This numerically higher difference in adherence between the two treatments compared to the overall analysis (3.8 percentage points) shows that the adherence advantage persists in more-experienced patients. Moreover, given that the advantage of rivaroxaban over apixaban was numerically larger among prior OAC users compared to the overall analysis, the results suggest that future research is needed to test whether patients more experienced with NVAF management, and with potentially higher motivation to take the treatment as prescribed, would benefit more from the simplified rivaroxaban dosing regimen. Nonetheless, the results of the present study are promising for reducing the risk of stroke in NVAF patients considering that it has previously been suggested that a 3% to 6% percentage point change in adherence is likely to be significant among patients with high-risk conditions. [25]

In the present study, a number of factors were associated with decreased adherence. For example, presence of multiple comorbidities decreased adherence among prior OAC users regardless of the drug, but adherence to therapy was still significantly better with rivaroxaban (by 5.6 percentage points). This finding may be explained by a higher pill burden among sicker patients. It has been highlighted in the literature that patients with high pill burden are at greater risk of being non adherent to therapy. [26,27] Therefore, once-daily medication could be a positive option to reduce the pill burden of patients and potentially increase adherence to NOAC agents among this specific subset of patients. These studies corroborated results of the present study suggesting that patients using a once-daily medication, such as rivaroxaban, are more adherent than patients using twice-daily medication with higher impact in specific subgroups of patients.

Previous work has examined how specific non-adherence risk factors for NVAF (e.g., age and experience managing other medical conditions [e.g., hypertension and diabetes]) affect adherence to NOAC therapy. [16] In this study, the presence of impaired cognitive/functional ability to take oral medications decreased adherence among prior OAC users regardless of the drug although adherence again remained significantly higher with rivaroxaban (by 3.2 percentage points). For patients with these impaired abilities, adherence may be higher for once-daily NOACs compared to twice-daily NOACs because the lower pill burden of a single dose may be less taxing given the patients’ cognitive/functional impairments. Given that once- and twice-daily NOACs were found to be similarly effective in terms of stroke prevention in recent studies [28,29], higher adherence to once-daily NOACs should be associated with superior protection for patients compared to lower adherence to twice-daily NOACs. However, twice-daily NOACs may be more forgiving for non-adherent patients. [30]

This study has some limitations. First, results reflect only adherence during the first 6 months of therapy among newly-initiated patients who used the therapy for at least 6 months, which gives no insights on longer term adherence (i.e., beyond 6 months). Second, results reflect the implementation phase of adherence and do not address the initiation and discontinuation phases; the latter might be an important type of non-adherence. [30,31] Third, the measure of adherence relied on dispensed prescriptions and days of supply from each dispensing assuming that all pills supplied were taken. Since it is not possible to confirm the pill intake with pharmacy claims data, this could result in overestimated adherence rates for both rivaroxaban and apixaban patients. Furthermore, the measure of adherence used does not capture adherent behavior by patients who do not submit insurance claims (i.e., patients who pay out of pocket). Fourth, the PQA measure used in this study may overestimate adherence given that the method requires patients to have at least two dispensings. Moreover, the method does not detect physician-recommended cessation of therapy. Fifth, claims data often does not capture anticoagulation treatment during inpatient stays which can lead to underestimation of adherence (especially in patients with long inpatient stays). Sixth, despite generally complete and accurate information in Truven databases, coding inaccuracies and missing data remain a possibility. Of note, there may also be differences in the likelihood of errors for primary diagnoses vs. secondary diagnoses (e.g., the ICD codes used in the present study for the propensity-based matching analysis). However, it is unlikely for these errors to affect the two treatment cohorts differently or to have any substantial impact on the results given the large sample sizes. Finally, a common limitation of observational studies is that adjustments can only be made for observed variables, and omitted- variable bias may still exist. Despite this, retrospective, observational studies that control for confounding factors through matching techniques provide quality results generalizable to real-life scenarios.

## Conclusion

In this large retrospective claims study, rivaroxaban users had better adherence to treatment at 6 months compared to apixaban users. The advantage for rivaroxaban over apixaban manifested itself both overall and among prior OAC users. Factors such as multiple comorbidities and non-adherence risk-factors decreased adherence in prior OAC users yet the adherence advantage of rivaroxaban was preserved even in these complicated to treat sub-populations.

## Supporting information

S1 Table ICD-9 codes for non-adherence risk factors. (DOCX)
