# Supplementary material for: Adherence to rivaroxaban versus apixaban among patients with non-valvular atrial fibrillation: Analysis of overall population and subgroups of prior oral anticoagulant users
Source: PLoS One. 2018 Apr 5;13(4):e0194099. doi: 10.1371/journal.pone.0194099 (PMC5886396; doi:10.1371/journal.pone.0194099)
Supplement: S1 Table — (DOCX) [file pone.0194099.s001.docx]

**S1 Table. ICD-9 Codes for Non-Adherence Risk Factors**
